# Supplementary material for: Characterisation of Waterborne Psychrophilic Massilia Isolates with Violacein Production and Description of Massilia antarctica sp. nov
Source: Microorganisms. 2022 Mar 24;10(4):704. doi: 10.3390/microorganisms10040704 (PMC9028926; doi:10.3390/microorganisms10040704)
Supplement: Supplementary file 1 [file microorganisms-10-00704-s001.zip › microorganisms-1631156-supplementary.pdf]

# **Characterisation of waterborne psychrophilic *Massilia* isolates with violacein production and description of *Massilia antarctica* sp. nov.**

Ivo Sedláček[a,*], Pavla Holochová[a], Hans-Jürgen Busse[b], Vendula Koublová[a], Stanislava Králová[a], Pavel Švec[a], Roman Sobotka[c], Eva Staňková[a], Jan Pilný[c], Ondrej Šedo[d], Jana Smolíková[e] and Karel Sedlář[f,g]

[a] Department of Experimental Biology, Czech Collection of Microorganisms, Faculty of Science, Masaryk University, Kamenice 5, 625 00 Brno, Czech Republic; ivo@sci.muni.cz (I.S.); pavlah@sci.muni.cz (P.H.); 436839@mail.muni.cz (V.K.); kralova.s@sci.muni.cz (S.K.); evickakroupova@seznam.cz (E.S.); mpavel@sci.muni.cz (P.Š.)

[b] Institut für Mikrobiologie, Veterinärmedizinische Universität Wien, Veterinärplatz 1, A-1210 Wien, Austria; hans-juergen.busse@web.de

[c] Centrum Algatech, MBÚ AV ČR, Novohradská 237 – Opatovický mlýn, 379 01 Třeboň, Czech Republic; Sobotka@alga.cz (R.S.); pilny@alga.cz (J.P.)

[d] Central European Institute of Technology, Masaryk University, Kamenice 5, 625 00 Brno, Czech Republic; sedo@post.cz

[e] Department of Physical Geography and Geoecology, Faculty of Science, Charles University, Albertov 6, 128 00 Praha 2, Czech Republic; janca.smolikova@gmail.com

[f] Department of Biomedical Engineering, Faculty of Electrical Engineering and Communication, Brno University of Technology, Technická 12, 616 00 Brno, Czech Republic; sedlar@vut.cz

[g] Institute of Bioinformatics, Department of Informatics, Ludwig-Maximilians-Universität München, Amalienstraße 17, 803 33 Munich, Germany; sedlar@bio.ifi.lmu.de

***Correspondence**: ivo@sci.muni.cz; Tel.: +420-549-496-922

## **Supplementary Materials.**

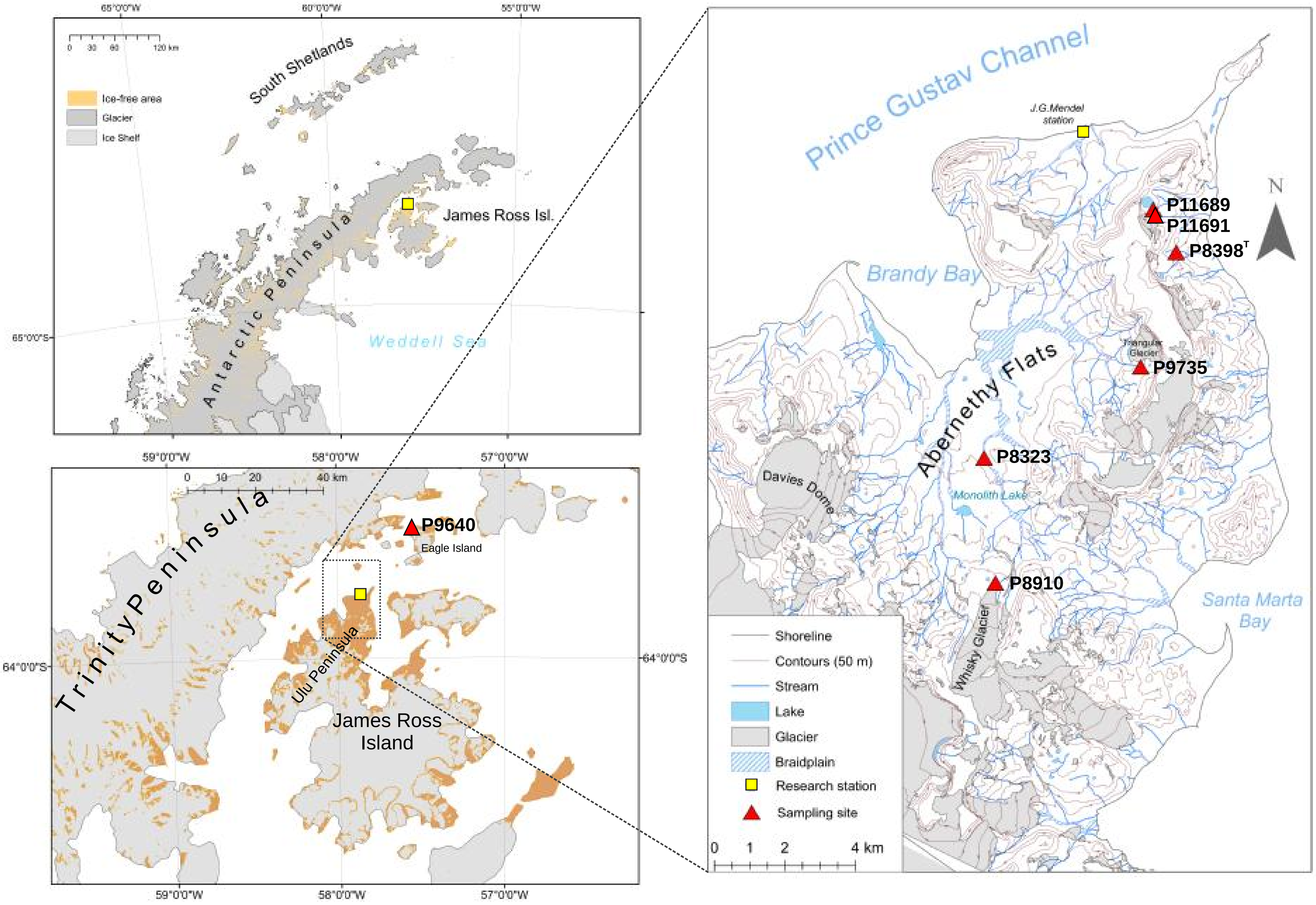


**Figure S1.** Sampling sites of *Massilia antarctica* sp. nov. isolates.

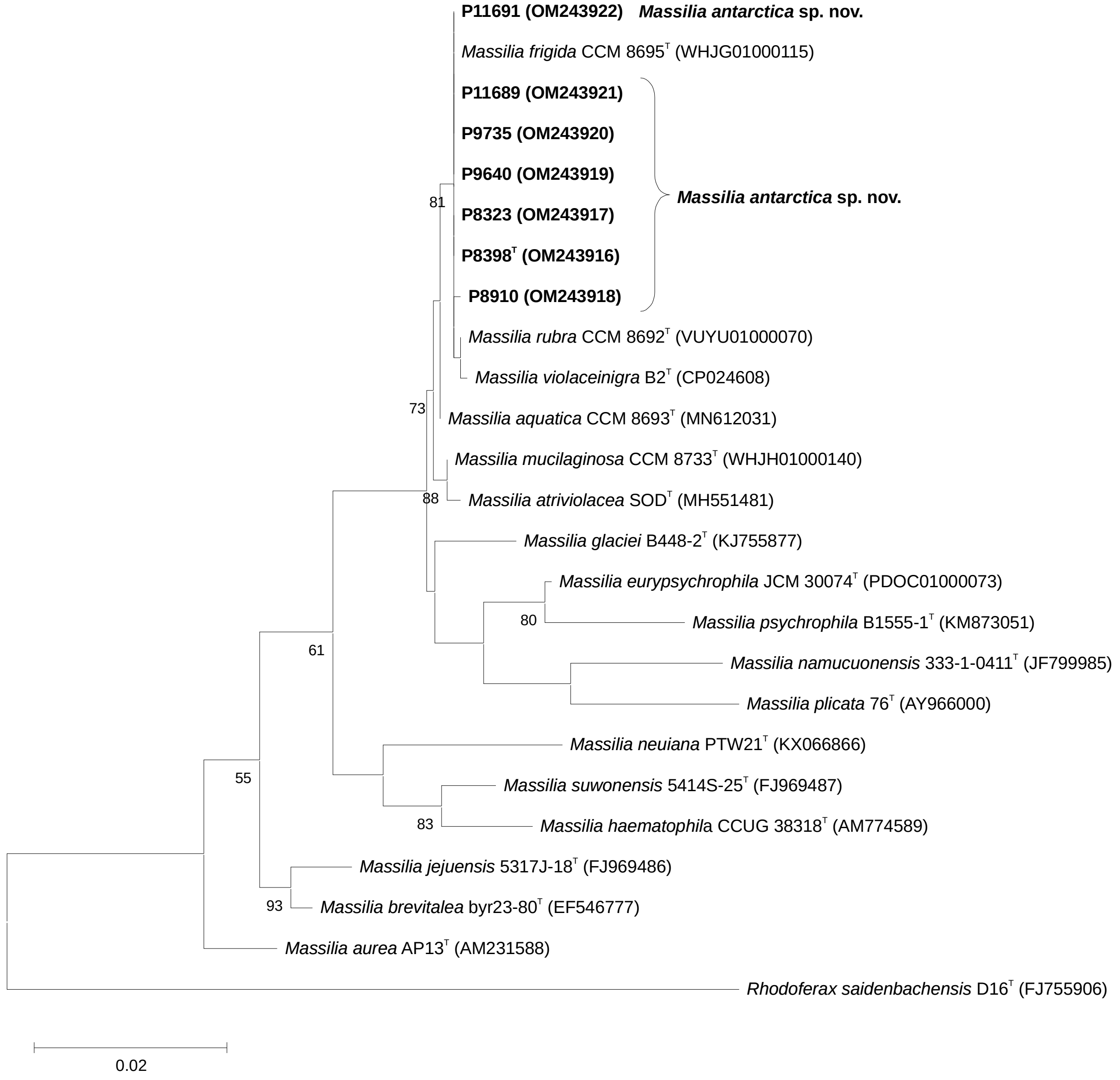


**Figure S2.** Maximum Likelihood phylogenetic tree based on 16S rRNA gene analysis showing the phylogenetic positions of the *M. antarctica* sp. nov. isolates and closely related *Massilia* ssp. type strains. Bootstrap probability values (percentages of 500 tree replications) greater than 50 % in which the associated taxa clustered together is shown next to the branches. The tree is drawn to scale, with branch lengths measured in the number of substitutions per site. *Rhodoferax saidenbachensis* D16$^{T}$ sequences were used as an outgroup. The GenBank accession numbers of the sequences are shown in parentheses. Bar, 0.02 substitutions per nucleotide position.

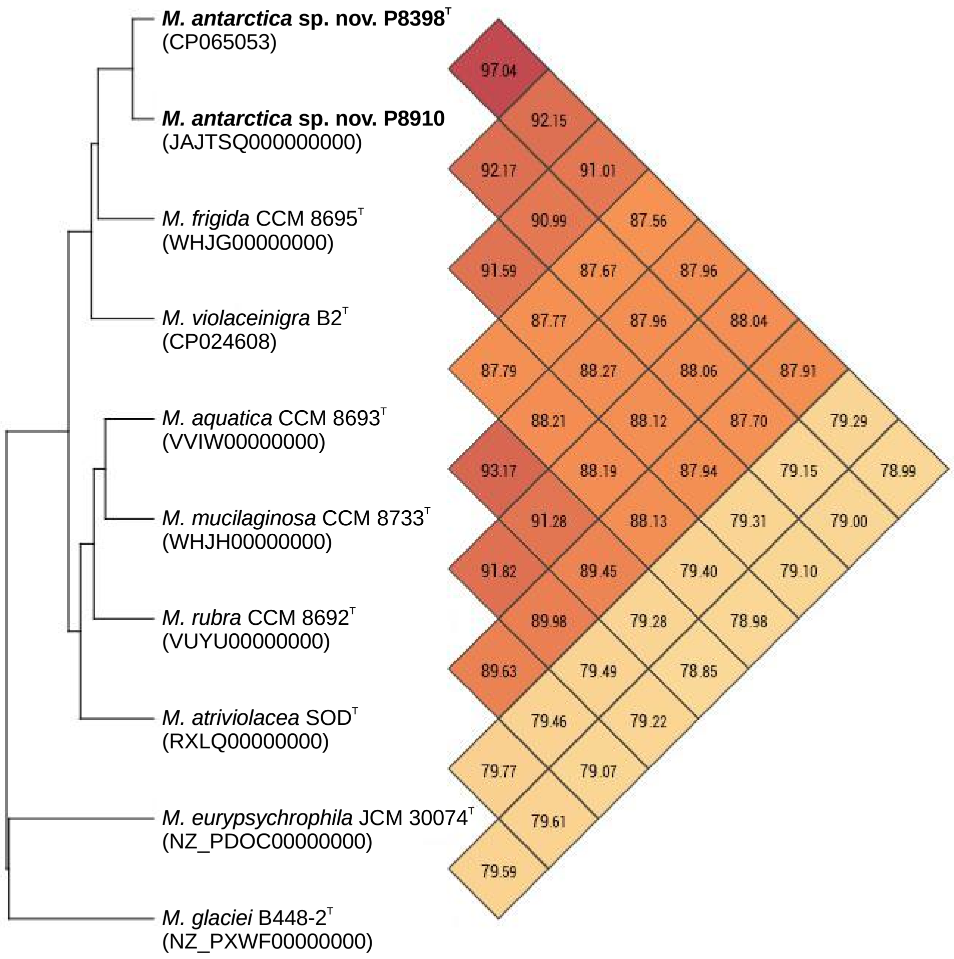


**Figure S3.** Heatmap generated with OrthoANI values obtained with the OAT software between the whole-genome sequences of *Massilia antarctica* sp. nov. P8398ᵀ and P8910 and the type strains of closely related *Massilia* spp. The accession numbers of the respective whole genome sequences are given in parentheses.

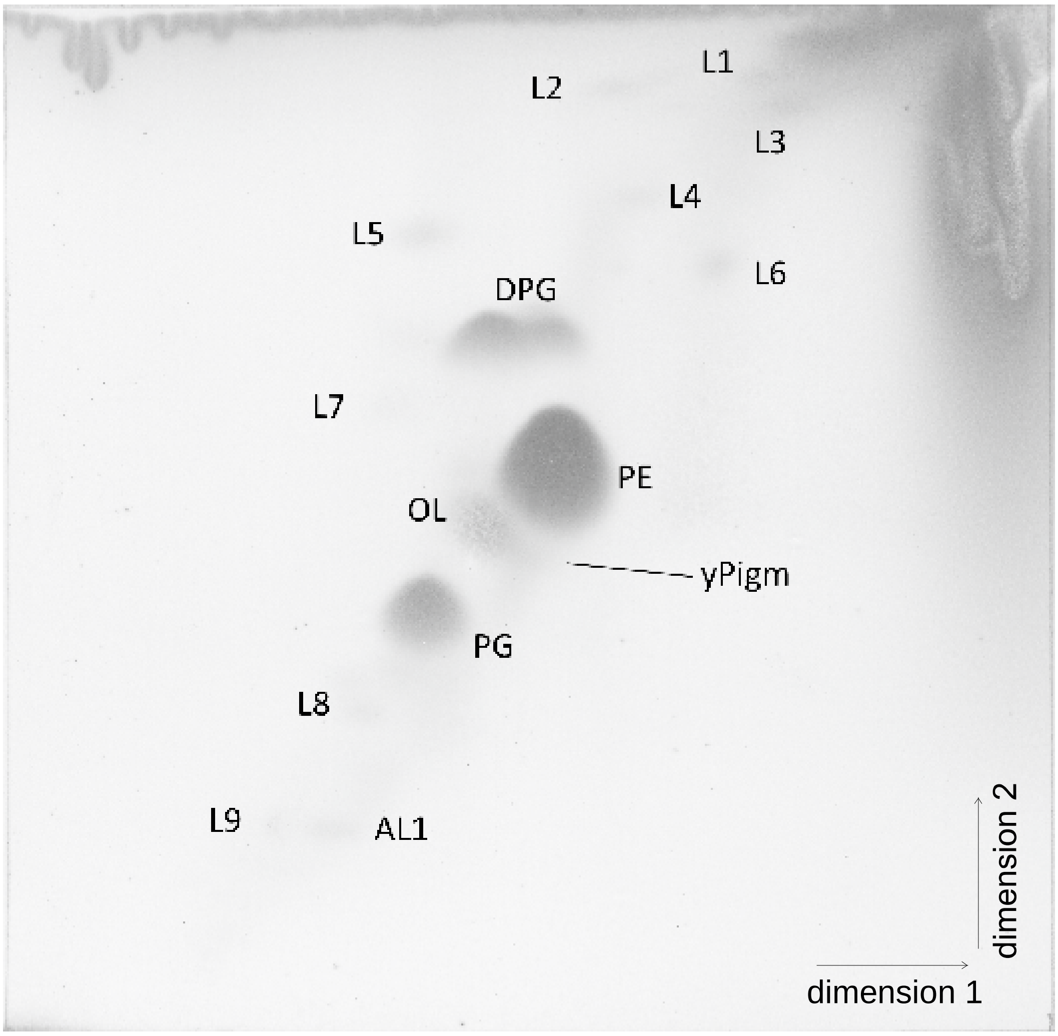


**Figure S4.** Polar lipid profile of strain P8398$^{T}$ after two-dimensional thin layer chromatography, detection with 5 % ethanolic molybdatophosphoric acid at 140 °C. Abbreviations: PE, phosphatidylethanolamine; PG, phosphatidylglycerol; DPG, diphosphatidylglycerol; OL, ornithine lipid; AL1, unidentified aminolipid; L1-9, unidentified polar lipids lacking a functional group; yPigm, yellow pigment.

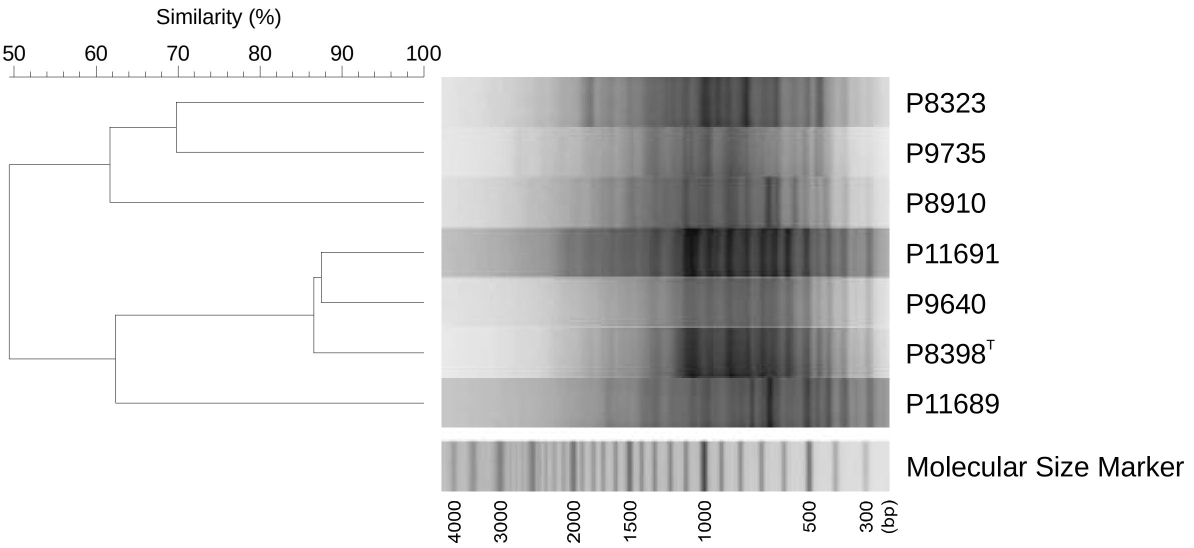


**Figure S5.** Dendrogram based on cluster analysis of rep-PCR fingerprints obtained with $(GTG)_5$ primer from *Massilia antarctica* sp. nov. strains. The dendrogram was calculated with Pearson's correlation coefficients with UPGMA clustering method (*r*, expressed as percentage similarity values).

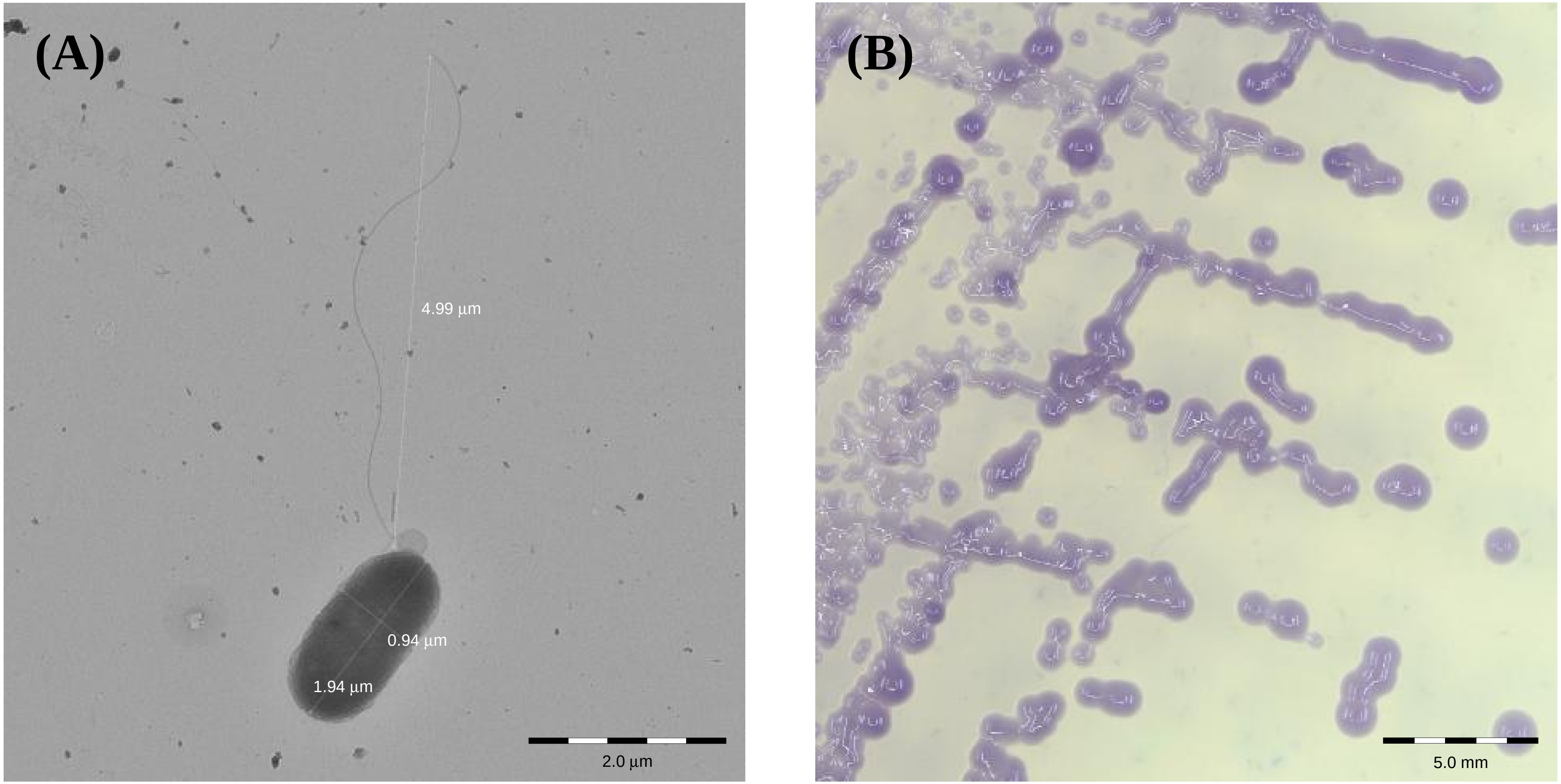


**Figure S6.** Morphology of strain P8398$^{T}$. (**A**) Cell morphology observed by transmission electron microscopy with a Philips Morgagni 268D electron microscope (FEI) using negative staining with 1% ammonium molybdate (original magnification ×6 000). (**B**) Colony morphology on R2A agar after 72 hours of cultivation at 20°C.

**Table S1.** Pairwise 16S rRNA gene sequence similarity values (%) of the *Massilia antarctica* sp. nov strains; 16S rRNA sequence similarity values with types of closely related *Massilia* spp. The GenBank accession numbers (16S rRNA, *gyr* B and *lep* A genes) are listed in the table. All data were taken from this study.

| ***Massilia antarctica*** sp. nov | ***Massilia antarctica*** sp. nov. | | | | | | | *M. rubra* | *M. aquatica* | *M. mucilaginosa* | *M. frigida* | *M. violaceinigra* | *M. atriviolacea* | *M. glaciei* | *M. eurypsychrophila* |
|---|---|---|---|---|---|---|---|---|---|---|---|---|---|---|---|
| | P8323 | P8398$^{T}$ | P8910 | P9640 | P9735 | P11689 | P11691 | CCM 8692$^{T}$ | CCM 8693$^{T}$ | CCM 8733$^{T}$ | CCM 8695$^{T}$ | CCM 8877$^{T}$ | CCM 8999$^{T}$ | CCM 8861$^{T}$ | CCM 8735$^{T}$ |
| P8323 | 100.0 | | | | | | | 99.8 | 99.7 | 99.5 | 99.9 | 99.7 | 99.4 | 98.8 | 98.6 |
| P8398$^{T}$ | 100.0 | 100.0 | | | | | | 99.9 | 99.9 | 99.7 | 100 | 99.7 | 99.4 | 98.8 | 98.6 |
| P8910 | 99.7 | 99.7 | 100.0 | | | | | 99.8 | 99.7 | 99.5 | 99.9 | 99.9 | 99.5 | 98.9 | 98.8 |
| P9640 | 100.0 | 100.0 | 99.9 | 100.0 | | | | 99.9 | 99.9 | 99.7 | 100 | 99.9 | 99.5 | 98.9 | 98.7 |
| P9735 | 100.0 | 100.0 | 99.9 | 100.0 | 100.0 | | | 99.9 | 99.9 | 99.7 | 100 | 99.7 | 99.4 | 98.8 | 98.6 |
| P11689 | 99.8 | 99.8 | 99.8 | 99.9 | 99.9 | 100.0 | | 99.9 | 99.8 | 99.6 | 99.9 | 99.8 | 99.5 | 98.9 | 98.8 |
| P11691 | 100.0 | 100.0 | 99.9 | 100.0 | 100.0 | 99.9 | 100.0 | 99.9 | 99.9 | 99.7 | 100 | 99.9 | 99.5 | 99.0 | 98.8 |
| **16S rRNA accession no.** | OM243917 | OM243916 | OM243918 | OM243919 | OM243920 | OM243921 | OM243922 | MN611986 | MN612031 | MN612043 | MN612047 | KF267246 | MH551481 | KJ755877 | KJ361504 |
| ***gyr* B accession no.** | OM296117 | OM296116 | OM296118 | OM296119 | OM296120 | OM296121 | OM 296122 | MN600847 | MN600892 | MN600904 | MN600908 | CP024608 | RXL.Q01000 007 | PXWF02000 255 | PDOC010000 01 |
| ***lep* A accession no.** | OM296124 | OM296123 | OM296125 | OM296126 | OM296127 | OM296128 | OM296129 | MN600911 | MN600956 | MN600968 | MN600972 | CP024608 | RXL.Q01000 008 | PXWF02000 285 | PDOC010000 03 |

**Table S2.** Clusters of orthologous groups of *Massilia antarctica* sp. nov. P8398$^{T}$

| COG class | Description | Gene count | Percentage |
|---|---|---|---|
| A | RNA processing and modification | 2 | 0.03 |
| B | Chromatin structure and dynamics | 3 | 0.05 |
| C | Energy production and conversion | 281 | 4.40 |
| D | Cell cycle control, cell division, chromosome partitioning | 59 | 0.92 |
| E | Amino Acid transport and metabolism | 374 | 5.85 |
| F | Nucleotide transport and metabolism | 113 | 1.77 |
| G | Carbohydrate transport and metabolism | 224 | 3.50 |
| H | Coenzyme transport and metabolism | 188 | 2.94 |
| I | Lipid transport and metabolism | 202 | 3.16 |
| J | Translation, ribosomal function and biogenesis | 207 | 3.24 |
| K | Transcription | 413 | 6.46 |
| L | Replication, recombination and repair | 255 | 3.99 |
| M | Cell wall/membrane/envelope biogenesis | 335 | 5.24 |
| N | Cell motility | 244 | 3.82 |
| O | Posttranslational modification, protein turnover, chaperones | 184 | 2.88 |
| P | Inorganic ion transport and metabolism | 227 | 3.55 |
| Q | Secondary metabolites biosynthesis, transport and catabolism | 93 | 1.45 |
| S | Function Unknown | 1099 | 17.19 |
| T | Signal transduction mechanisms | 351 | 5.49 |
| U | Intracellular trafficking, secretion, and vesicular transport | 121 | 1.89 |
| V | Defense mechanisms | 80 | 1.25 |
| | COG unknown | 1337 | 20.92 |

**Table S3.** Phage DNA within the *Massilia antarctica* sp. nov. P8398$^{T}$ genome

| Position | Length [kbp] | Status | Total no. of genes |
|---|---|---|---|
| 964,028-978,623 | 14.5 | questionable | 17 |
| 3,590,109-3,629,201 | 39 | intact | 54 |

**Table S4.** R-M systems in the *Massilia antarctica* sp. nov. P8398$^T$ genome

| Type | Name | Locus Tag | Gene |
|---|---|---|---|
| I | M.Msp8941ORF10495P | IV454_10495 | Modification |
| I | S1.Msp8941ORF10495P | IV454_10500 | Specificity |
| I | S2.Msp8941ORF10495P | IV454_10505 | Specificity |
| I | Msp8941ORF10495P | IV454_10510 | Restriction |
| II | M.Msp8941ORF6205P | IV454_06205 | Modification |
| II | M.Msp8941ORF6925P | IV454_06925 | Modification |
| IV | Msp8941ORF6245P | IV454_06245 | Restriction |

**Table S5.** Cellular fatty acid contents (%) of *Massilia antarctica* sp. nov. strains and the closest phylogenetic relatives (*Massilia atriviolacea* CCM 8999$^{T}$, *Massilia violaceinigra* CCM 8877$^{T}$, *Massilia rubra* CCM 8692$^{T}$, *Massilia mucilaginosa* CCM 8733$^{T}$, *Massilia aquatica* CCM 8693$^{T}$, *Massilia frigida* CCM 8735T, *Massilia glaciei* CCM 8861$^{T}$, *Massilia eurypsychrophila* CCM 8735$^{T}$).

All data were taken from this study using cells grown to the late exponencial phase (72 h) on R2A medium at 20 °C. TR, traces (< 1.0%); ND, not detected.

| **Strain No.** / **Fatty acid** | P8323 | P8398$^{T}$ | P8910 | P9640 | P9735 | P11689 | P11691 | CCM 8999$^{T}$ | CCM 8877$^{T}$ | CCM 8692$^{T}$ | CCM 8733$^{T}$ | CCM 8693$^{T}$ | CCM 8695$^{T}$ | CCM 8861$^{T}$ | CCM 8735$^{T}$ |
|---|---|---|---|---|---|---|---|---|---|---|---|---|---|---|---|
| $C_{10:0}$ | 3.6 | 3.3 | 3.2 | 3.4 | 4.0 | 3.7 | 3.2 | ND | TR | ND | ND | ND | TR | TR | TR |
| $C_{10:0}$ 3OH | 3.8 | 3.5 | 3.4 | 3.4 | 3.6 | 3.1 | 3.2 | 2.8 | 2.1 | 2.7 | 2.9 | 2.4 | 2.0 | 6.5 | 2.0 |
| $C_{12:0}$ | 5.1 | 4.7 | 4.7 | 4.9 | 5.9 | 5.4 | 4.8 | 4.9 | 4.8 | 3.6 | 4.2 | 3.5 | 2.7 | 3.5 | 2.7 |
| $C_{12:0\ 3OH}$ | 3.8 | 4.0 | 3.7 | 4.0 | 3.9 | 3.5 | 3.6 | 3.5 | 2.0 | 3.4 | 3.6 | 3.2 | ND | ND | ND |
| $C_{14:0}$ | TR | ND | ND | ND | 1.0 | TR | TR | TR | TR | ND | ND | TR | TR | TR | TR |
| $C_{16:0}$ | 27.1 | 24.1 | 26.3 | 25.0 | 24.7 | 22.9 | 23.4 | 24.7 | 24.6 | 25.0 | 23.2 | 24.1 | 20.4 | 22.3 | 20.4 |
| $C_{18:0}$ | TR | ND | ND | ND | ND | ND | TR | ND | TR | TR | ND | 1.2 | TR | 1.0 | TR |
| *Summed feature 3 | 52.4 | 56.1 | 53.9 | 55.6 | 57.1 | 56.7 | 56.0 | 58.0 | 60.9 | 59.4 | 57.8 | 59.6 | 61.3 | 61.9 | 61.3 |
| *Summed feature 8 | 3.0 | 4.3 | 4.8 | 3.9 | ND | 3.4 | 4.7 | 5.5 | 4.4 | 5.2 | 7.8 | 5.5 | 8.7 | 1.0 | 8.7 |

*Summed features are groups of two fatty acids that cannot be separated by gas chromatography using the MIDI system.
Summed feature 3 contains $C_{16:1}\omega 7c$ /$C_{16:1}\omega 6c$ and Summed feature 8 contains $C_{18:1}\omega 7c$ /$C_{18:1}$iso $\omega 6c$ .

**Table S6.** Variable reactions of *Massilia antarctica* sp. nov. strains (plate/tube tests, API ZYM kit, Biolog GEN III system).

| Test | P8323 | P8398[T] | P8910 | P9640 | P9735 | P11689 | P11691 |
|---|---|---|---|---|---|---|---|
| Nitrate reduction | + | + | - | - | - | + | + |
| Hydrolysis of: DNA | + | + | - | - | - | + | + |
| lecithine | + | - | + | + | + | - | - |
| Acid from mannitol | + | - | + | - | + | - | - |
| Ampicillin (10 µg) | S | R | S | S | S | R | R |
| Carbenicillin (100 µg) | S | R | S | S | S | R | S |
| Ceftazidim (10 µg) | S | R | S | S | S | S | R |
| API ZYM: esterase | + | + | - | - | - | - | - |
| valine arylamidase | + | + | + | + | + | + | - |
| trypsin | - | + | - | - | - | - | - |
| acid phosphatase | w | + | w | + | - | - | - |
| naphtol-AS-BI-phospohydrolase | + | + | w | - | - | - | - |
| α-glucosidase | - | + | + | + | + | - | - |
| β-glucosidase | - | + | w | - | - | - | - |
| Biolog GEN III: dextrin | - | - | - | b | - | - | - |
| D-cellobiose | - | b | - | - | - | - | - |
| D-fructose-6 PO4 | - | - | b | b | b | + | - |
| D-glucose-6 PO4 | - | - | - | - | - | - | + |
| L-serine | - | - | - | - | - | - | w |
| glucuronamide | - | - | - | b | - | - | - |
| quinic acid | - | - | - | - | - | + | - |
| L-malic acid | - | b | - | - | + | + | - |
| β-hydroxy-D,L-butyric acid | b | b | b | - | + | - | - |
| α-keto butyric acid | b | - | b | b | - | + | - |
| acetoacetic acid | - | - | - | b | - | - | - |

All data were taken from this study. +, positive; w, weak; b, borderline; -, negative; S, sensitive; R, resistant.
